# C_4_ Photosynthesis Promoted Species Diversification during the Miocene Grassland Expansion

**DOI:** 10.1371/journal.pone.0097722

**Published:** 2014-05-16

**Authors:** Elizabeth L. Spriggs, Pascal-Antoine Christin, Erika J. Edwards

**Affiliations:** Department of Ecology and Evolutionary Biology, Brown University, Providence, Rhode Island, United States of America; University of Warwick, United Kingdom

## Abstract

Identifying how organismal attributes and environmental change affect lineage diversification is essential to our understanding of biodiversity. With the largest phylogeny yet compiled for grasses, we present an example of a key physiological innovation that promoted high diversification rates. C_4_ photosynthesis, a complex suite of traits that improves photosynthetic efficiency under conditions of drought, high temperatures, and low atmospheric CO_2_, has evolved repeatedly in one lineage of grasses and was consistently associated with elevated diversification rates. In most cases there was a significant lag time between the origin of the pathway and subsequent radiations, suggesting that the ‘C_4_ effect’ is complex and derives from the interplay of the C_4_ syndrome with other factors. We also identified comparable radiations occurring during the same time period in C_3_ Pooid grasses, a diverse, cold-adapted grassland lineage that has never evolved C_4_ photosynthesis. The mid to late Miocene was an especially important period of both C_3_ and C_4_ grass diversification, coincident with the global development of extensive, open biomes in both warm and cool climates. As is likely true for most “key innovations”, the C_4_ effect is context dependent and only relevant within a particular organismal background and when particular ecological opportunities became available.

## Introduction

Within flowering plants, the grasses (Poaceae) are a remarkable clade, in terms of both species richness and ecological breadth. Comprising over 11,000 species, grasses are exceptionally diverse and a dominant feature of most open habitats throughout the world. Although many share a common morphological form, important physiological differences define various groups of grasses and act to sort these into environmental types.

**Figure 1:**
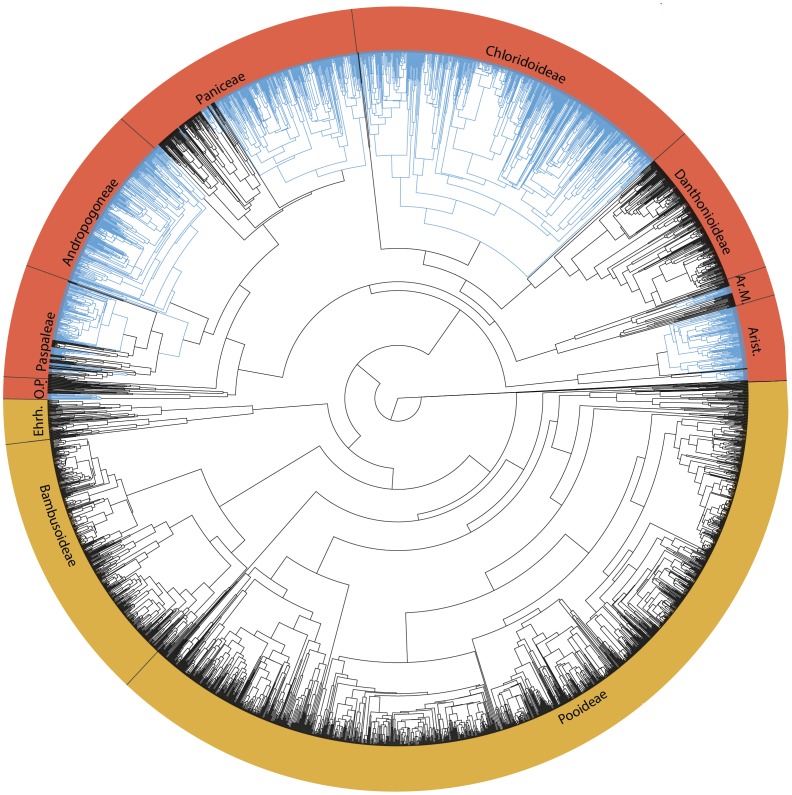
Poaceae phylogeny with 3595 taxa. C_4_ lineages are mapped in blue. Red labels indicate the PACMAD clade, yellow labels indicate the BEP clade, and grey labels indicate the early diverging Poaceae lineages. Lineage names are abbreviated as: O.P. Outlying Panicoideae, Ehrh. Ehrhartoideae, Ar.M. Arundinoideae+Micrairoideae, and Arist. Aristidoideae.

Grasses living in tropical and subtropical grassland or savanna systems almost exclusively utilize the C_4_ photosynthetic pathway [1]–[3]. This trait is a complex modification over the ancestral C_3_ pathway that confers an advantage in open, hot, and dry conditions by concentrating CO_2_ inside plant cells and preventing high levels of photorespiration [4]. C_4_ photosynthesis characterizes several ecologically dominant, species-rich lineages, suggesting that the C_4_ trait may also promote lineage diversification, via either a reduction in extinction rate, an increase in speciation rate, or a combination of both. In the past decade, molecular phylogenies have revealed the existence of three species-poor grass lineages successively sister to the rest of Poaceae and have placed the bulk of grass diversity in either the BEP or PACMAD clade (Figure 1) [5]–[10]. All of the 22–24 C_4_ origins occur within the PACMAD clade, while the similarly sized BEP is entirely C_3_ [5]–[10]. This clustering of all C_4_ origins in one of the two major grass lineages may be partly due to increased evolutionary accessibility to the C_4_ trait in this clade, based on a shared set of leaf anatomical attributes [11].

In this study, we use phylogenetic comparative methods on large datasets to test for the effect of C_4_ photosynthesis on diversification rates within grasses. While a densely sampled phylogeny of the entire grass lineage is central to accurately identifying shifts in diversification, most previous phylogenetic efforts have concentrated on relatively small subgroups, with the result that few markers are consistently sampled throughout the lineage, and many are difficult to align across distantly related taxa [[*e.g.*12]–[20]]. Previous investigations of grass diversification rates have been hindered by this data structure and have included molecular data for less than 5% of grass diversity [21], [22]. To incorporate as many species as possible without introducing large amounts of missing data into the sequence alignments, we constructed 14 separate phylogenies, corresponding to the main lineages inside grasses, and each built with a unique, optimal set of markers. Using a well-resolved backbone phylogeny [10], these were combined into a set of trees that contained 3,595 taxa (Figure 1), encompassing about 30% of the estimated diversity in Poaceae [23]. Using these phylogenies, we found a strong and significant effect of C_4_ photosynthesis on diversification. We also explored these trees to identify shifts in diversification independently of any character state information, and interpret these analyses jointly, in the context of C_4_ evolution and Miocene grassland expansion.

## Materials and Methods

### Sequence Mining and Matrix Assembly

The majority of recent phylogenetic work in Poaceae has focused on specific subfamilies or genera and has employed a variety of fast-evolving chloroplast and nuclear markers (e.g. [12]–[20]). The nature of these studies has resulted in a wealth of sequence data for Poaceae, but many markers are both poorly sampled across the entire group and difficult to align across the entire clade. To circumvent the phylogenetic problems that arise from such data, specifically poor alignments, large amounts of missing sites, and large matrices ill-suited to computationally intensive analyses, we subdivided the tree-building approach. Fourteen sub-trees were constructed separately and subsequently inserted into a fossil-calibrated backbone phylogeny. This approach relies heavily on recent work in the grasses that has resolved deep relationships among the subfamilies and clarified discrepancies in various molecular dating efforts [6]–[8], [10], [24].

Sequence data was collected from Genbank with the PHLAWD tool (http://phlawd.net/ [25]) using the plant GenBank database generated in March 12, 2012. To avoid synonymy problems, all genus names were transformed to those accepted by the Kew taxonomic database, using the GrassBase [23] synonymy database. Because the taxonomic classification in Genbank is not consistent with the latest developments in grass taxonomy, clades based on GenBank names are not always monophyletic. Species were, therefore, sorted into groups based on previous studies [10] and inspected on preliminary phylogenetic trees as necessary. In general, monophyletic groups were defined to correspond to traditionally recognized clades. The Bambusoideae, Ehrhartoideae, Chloridoideae, Danthonioideae, Andropogoneae, Paspaleae, and Paniceae were all used. The species-poor sister clades Arundinoideae and Micrairoideae were combined, as were the outlying Panicoideae *sensu* GPWGII 2012 [10]. The Pooideae was too large to analyze in one piece, so after marker selection, 3 monophyletic clades were separated from the Pooideae backbone and each was analyzed individually. Two representatives of each separated clade were retained with the remaining backbone Pooideae so that their monophyly and divergence date could be constrained, and the separated lineage could be reinserted later. PHLAWD was then used to create alignments for the most frequently sampled gene regions in each of the 14 clades using a coverage threshold of 0.4 and an identity threshold of 0.1. The three plastid markers *matK, ndhF*, and *rbcL* were included in each group and an additional 2 to 10 gene regions were added depending on the group sampled (Table S2). In total, 35 gene regions were incorporated in the analysis (sampling information in Table S1, S2).

Once the alignments were complete, the software trimAl [26] was used to remove sites with more than 70% missing data for each gene region and the MEGA software [27] was used to manually edit the alignment where necessary. In each group, the alignments were concatenated with Phyutility [28] and species names were checked against the GrassBase [23] synonymy database. A small number of names were referenced in Tropicos [29] but not in GrassBase [23], and were consequently considered to be recently described species. Synonyms, misspellings, subspecies, and varieties were manually removed whenever possible to leave a single representative sequence per accepted species. At this point, RAxML software [30] was used to build a tree with 20 maximum likelihood searches, retaining the tree with the highest likelihood score across them. The phylogeny inferred for each group was manually inspected to identify taxa that had very long branches, representing potential errors. The sequences of these taxa were inspected by BLAST searches against GenBank, and putatively erroneous sequences, corresponding to either sequencing or identification errors, were removed.

### Tree Building and Molecular Dating

To estimate the age of the main grass lineages, dating analyses were first performed with a dataset of three previously sampled chloroplast genes and 543 taxa covering the entire grass family [10]. The software BEAST 1.7.2 [31] was run under a GTR+G+I substitution model, a Yule process for the prior distribution of node ages and a log-normal distribution for the prior on evolutionary rates among branches. Time-calibrated trees where obtained with two contrasting hypotheses for the placement of fossils [24]. Under calibration #1, which is based only on macrofossil calibrations and does not take into account fossil phytoliths whose placement is somewhat controversial [32], the crown age of the BEP-PACMAD clade followed a normal calibration density with a mean of 51.2 Ma and a standard deviation of 6.0 Ma [24]. Under calibration #2, which incorporates fossil phytoliths [32], the age of this same node followed a normal calibration density with a mean of 82.4 Ma and a standard deviation of 7.5 Ma [24]. In this second analysis, we also constrained the stem of Oryzeae to obtain dates compatible with phytolith fossil evidence [32], using an exponential distribution with a mean of 10 Ma offset by 67 Ma. For these two analyses, the topology was not fixed, except for the monophyly of the ingroup (all taxa except *Pharus*). Trees were sampled every 5,000 generations for 15,000,000 generations after a burn-in period of 5,000,000 generations. Convergence, effective sample size, and the adequacy of the burn-in period were assessed using Tracer 1.5 [31].

A phylogeny was then inferred separately for each previously defined group of grasses using the software BEAST as described above [31]. Crown node ages were fixed (uniform prior with range of 0.01 around the fixed value) to the dates obtained from the Bayesian consensus phylogeny estimated from the 543-taxon dataset (above), under calibration #1. All trees were then scaled to match the dates under calibration #2. All subsequent analyses were performed on both sets of time-calibrated phylogenetic trees. The monophyly of the ingroup was enforced to ensure proper rooting. For each dataset, two independent Markov Chain Monte Carlo analyses were run for 10–50 million generations, sampling every 1000–5000 generations, depending on the size of the dataset. Convergence, effective sample size, and the adequacy of the burn-in period were assessed using Tracer [31]. A burn-in period of 2,500,000–6,000,000 generations was chosen, again depending on the size of the dataset. For clades of over 150 taxa, convergence from random starting trees was extremely slow, and so the best of our previous 20 maximum likelihood RAxML trees was dated using non-parametric rate smoothing in r8s [33] and used as a starting point for each run.

For each group, the maximum clade credibility tree was selected with TreeAnnotator [31] and the node heights of this tree were scaled in R to match each of the dating hypotheses by multiplying all branch lengths by the fraction (hypothesis root age/current root age). The calibrated phylogenetic trees were then manually inserted into the associated backbone phylogeny of 543 grasses [10], preserving the deep relationships among the groups and forming a set of all-inclusive, ultrametric phylogenies with 3595 species each. With 544 genera represented, this tree contains more than 29% of the species and 71.2% of the recognized genera in Poaceae. Of the missing genera, only 6 have more than 10 species [23].

To take into account both phylogenetic uncertainty and variation in dating hypotheses, we repeated diversification analyses on 100 topologies drawn randomly from the population of trees sampled post burn-in by BEAST for each of our 14 groups. A unique, calibrated phylogeny for each group was scaled and added to each of our two backbone phylogenies to produce 100 alternative phylogenies of the grasses under each set of dating conditions.

### Diversification Analyses

Three approaches were used to analyze the patterns of diversification in Poaceae. First, the BiSSE (Binary State Speciation and Extinction) method [34], [35] specifically evaluated the relationship between photosynthetic type and diversification rate. Second, log-scale species richness was compared among sister groups with different photosynthetic types using a Wilcoxon sign ranked test [36]. Third, turboMEDUSA, a likelihood method implemented in R [37], was used to locate and quantify shifts in diversification rates across Poaceae independently of any character state information. Since all of the C_4_ origins occur within the PACMAD portion of Poaceae, our focus is on this clade, although we also ran analyses across the entire phylogeny.

To effectively evaluate diversification patterns, it was necessary to determine the richness and distribution of Poaceae species on our phylogeny. Although taxonomic issues remain unsettled in certain areas of Poaceae phylogeny, we were able to approximate the size of most genera using the accepted names in GrassBase [23]. Unless otherwise demonstrated, genera were assumed to be monophyletic and occasionally small genera nested in larger ones were merged. For each genus, the species with the most sequence data was selected as the representative of that group and was assigned the richness of the entire genus. In genera with both C_3_ and C_4_ taxa, we divided the genus into the minimum number of clades such that the C_3_ taxa and the C_4_ taxa were monophyletic and each represented by a single tip in our phylogeny. According to estimates from GrassBase [23], the phylogeny inferred in this study contains ∼25% of the known *Panicum* species. This genus is, however, highly polyphyletic [38], and diverse sections have been segregated into new genera in the past few years [38]–[44]. To cope with this uncertainty, the number of *Panicum* species was equally spread among all *Panicum* tips in our phylogeny and the well-supported monophyletic groups of *Panicum* were subsequently collapsed. Using this approach, we were able to assign 11,554 species (95.5% of Poaceae) to a specific tip on our tree [23].

An additional difficulty lay in the potential tendency for large clades on short branches to throw off diversification estimates. Therefore, large clades of over 190 species were split among several representatives. The genus *Poa*, for instance, which contains over 550 species, was divided evenly among the tips corresponding to *Poa pratensis*, *Poa annua*, and *Poa colensoi*. Even with similar subdivisions, excessively small state probabilities occasionally caused the BiSSE likelihood calculations to fail. In these cases, the groups were further subdivided among additional representatives or combined with a sister group to increase the subtending branch length.

Using TurboMEDUSA, the number of shifts in diversification rates was first estimated with the default AICc threshold on our genus level tree (8.4547). Each representative tip was assigned the same richness value used for the BiSSE analyses. This approach suggested 24 shifts, some of which were located on extremely short branches leading to a single tip, with a relatively small number of species. These shifts were no longer identified with a more conservative threshold of 10.5, which suggested 18 shifts in diversification. These shifts were considered more reliable and are reported here.

Pruning our large phylogeny down to single representatives of each genus allowed us to include information about unsampled diversity in our analyses, but it also reduced a substantial amount of branching structure and information. For example, using this approach with TurboMEDUSA precludes the identification of shifts that might occur closer to the tips, within genera for instance. We therefore performed a complimentary TurboMEDUSA analysis on the complete 3595 species tree. We also ran BiSSE analyses on the unpruned, 3595 tip tree, accommodating unsampled diversity by reporting our overall sampling frequency (0.2973 for Poaceae, and 0.2966 for PACMAD [23]) which BiSSE then used in calculations.

Tree inference, dating analyses, and diversification analyses were conducted on the OSCAR HPC cluster at Brown University and the Louise HPC cluster at Yale University. Sequence matrices, trees, and character matrices have all been deposited on dataDryad doi:10.5061/dryad.74b5d.

## Results

### BiSSE

Our BiSSE analyses provided extremely strong support for the evolution of C_4_ photosynthesis increasing diversification rates in grasses. All of the BiSSE tests on the BEAST maximum credibility tree strongly rejected the model of equal diversification rates for C_3_ and C_4_ taxa. This was irrespective of how we accommodated unsampled diversity, whether we analyzed PACMAD separately or together with all of Poaceae, and whether we calibrated our phylogeny with phytoliths or with less-controversial macrofossils (Table 1, Table S3). In most cases, the best-fitting model was a 6-parameter model in which both speciation and extinction rates were different for C_3_ and C_4_ taxa, but this was often only marginally better than models where C_3_ and C_4_ lineages differed only in either speciation or extinction rates. Regardless, equal diversification rates were soundly rejected, and in all cases, C_4_ diversification rates were inferred to be higher. This C_4_ effect can also clearly be seen in a linage-through-time plot (Figure S1).

**Table 1:** Parameters inferred for the PACMAD sampling frequency (proportional) tree.

Model	Parameters	Speciation Rates		Extinction Rates		Net Diversification Rates		Transition Rates		Ln Likelihood
**Dating Hypothesis 1**		C_3_	C_4_	C_3_	C_4_	C_3_	C_4_	To C_3_	To C_4_	
equal diversification	4	0.6122	0.6122	0.4076	0.4076	0.2047	0.2047	0.0104	0.0001	−4803.4520
**equal speciation**	**5**	**0.5991**	**0.5991**	**0.4488**	**0.3652**	**0.1503**	**0.2339**	**0.0083**	**0.0000**	**−4792.1430**
equal extinction	5	0.5600	0.6219	0.3963	0.3963	0.1637	0.2256	0.0095	0.0000	−4794.8060
**full model**	**6**	**0.6924**	**0.5667**	**0.5539**	**0.3267**	**0.1386**	**0.2400**	**0.0068**	**0.0001**	**−4790.4060**
**Dating Hypothesis 2**										
equal diversification	4	0.9383	0.9383	0.8718	0.8718	0.0665	0.0665	0.0046	0.0000	−5840.7290
**equal speciation**	**5**	**0.9300**	**0.9300**	**0.8962**	**0.8399**	**0.0338**	**0.0901**	**0.0038**	**0.0000**	**−5828.5740**
equal extinction	5	0.8404	0.8964	0.8051	0.8051	0.0353	0.0913	0.0039	0.0000	−5831.2130
**full model**	**6**	**0.3627**	**0.3011**	**0.2857**	**0.1667**	**0.0770**	**0.1344**	**0.0037**	**0.0001**	**−5827.0790**

Values as inferred under a 4-parameter model (speciation and extinction rates both constrained to be equal), two 5-parameter models (extinction and speciation rates allowed to differ in each respectively), and a 6-parameter model (separate speciation and extinction rates for C_3_ and C_4_ taxa).

**Figure 2:**
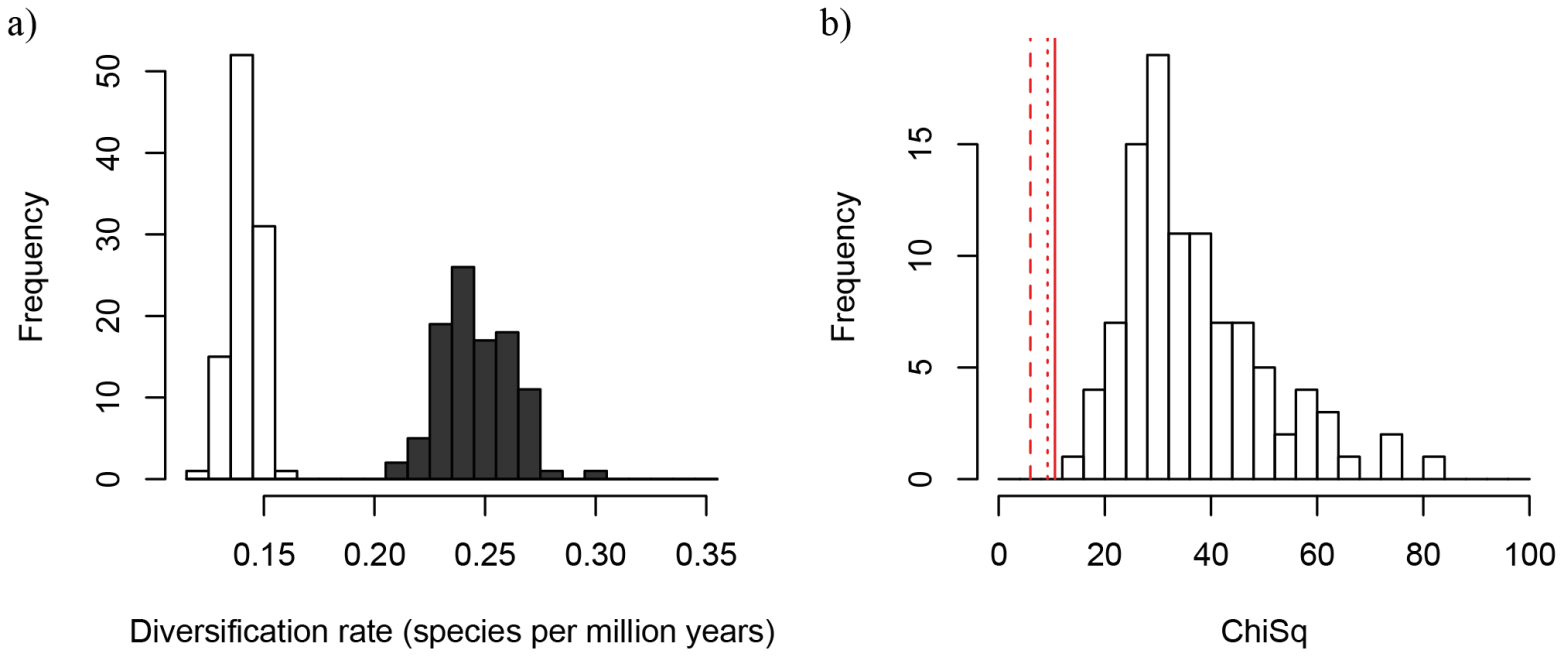
Histograms of BiSSE model inferences based on 100 replicate PACMAD trees. Each tree had 1774 taxa, and the missing diversity was represented as a proportion (sampling frequency). Black bars indicate C_4_ rates, white bars indicate C_3_ rates. The panels show: a. Net diversification rates derived from a 6-parameter model, b. Chi Squared values derived from ANOVA comparison of a 6-parameter model and a 4-parameter (equal diversification) model for each tree. The red lines indicate significance values of .05, .01, and .005.

The replicate BiSSE analyses on 100 trees from the posterior distribution indicate that these results are also robust to phylogenetic uncertainty. Across 100 replicate PACMAD trees, equal diversification rates were strongly rejected (p<0.01) in all but one tree regardless of whether the missing diversity was distributed proportionally or by genus (Figure 2; Figure S2). Poaceae-wide trees similarly provided additional support, but only when analyses were performed using the full 3,595 taxon tree and missing species were distributed evenly across the tips (Figure S2). Lack of support in these trees when diversity was distributed by genus is probably due to the extremely high numbers of species in C_3_ BEP genera like *Festuca*, *Poa*, and *Stipa* which were each clustered at a few tips in the genus-level analyses.

In the PACMAD clade, when the missing species diversity was distributed by genus, BiSSE estimated a net C_4_ diversification rate of 0.1458 spp/my and a net C_3_ rate of 0.0951 spp/my in the maximum credibility tree under the macrofossil-dating hypothesis (Table 1). When the missing species diversity was instead distributed proportionally, both diversification rates were estimated to be much higher (0.2407 spp/my for C_4_, 0.1677 spp/my for C_3_) (Table S3). When the entire Poaceae tree was used, the estimated C_3_ and C_4_ rates were very similar to those identified when using the PACMAD tree alone, with significantly higher C_4_ rates of diversification (p<.01; Table S3).

Under the phytolith-based dating calibration, the results from all analyses were consistent with those based on the macrofossil dates, with the obvious exception that actual net rates of diversification were estimated to be much lower, because grasses were inferred to be older. Similar contrasts between the C_3_ and C_4_ net diversification rates were evident, and models of equal diversification were rejected under the same conditions at similar levels of confidence (Table S3, Figure S2).

### Sister Group Comparisons

Nearly half of the C_4_ lineages in grasses are sister to groups that contain both C_3_ and C_4_ taxa; however, 12 have exclusively C_3_ sister clades and could be compared directly (Table S4). Of these, the C_4_ group is equally or more diverse in ten cases and the log-scaled species richness is significantly greater in C_4_ groups (Wilcoxon sign ranked test p-value  = 0.0067). While the independent C_4_ lineages differ greatly in both age and species richness, they are consistently more diverse than their C_3_ sisters (Table S4).

### turboMEDUSA

**Figure 3:**
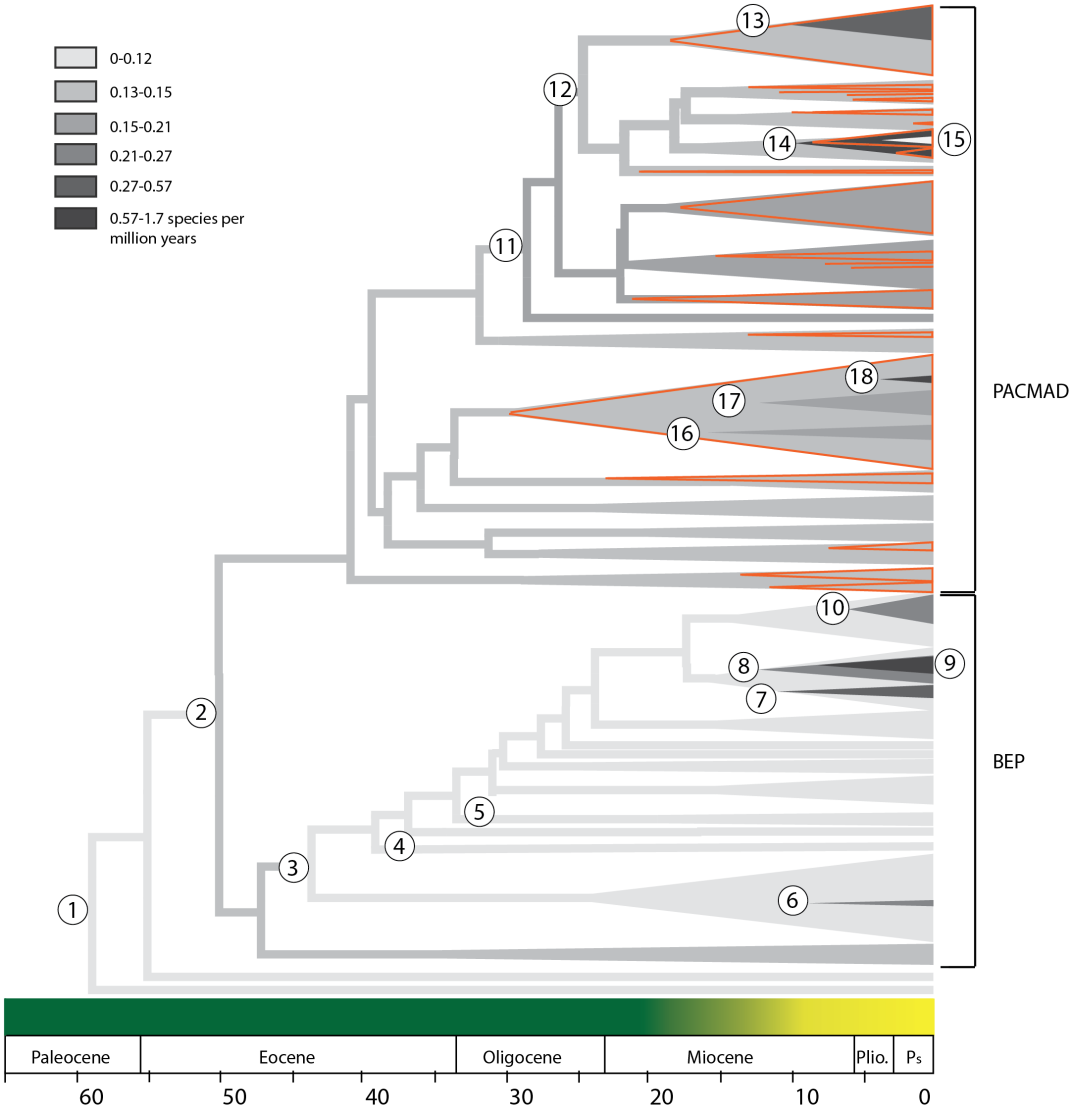
Simplified representation of shifts in diversification rates across Poaceae based on calibration #1. Darker shades of grey indicate higher rates of diversification. Red triangles indicate the approximate phylogenetic placement of C_4_ lineages. The left point of each triangle corresponds to the stem age of the inferred shift. The transition from dark green to yellow across the bottom indicates the average timing of the rise of open, grassland habitats on different continents [2]. Rate shifts correspond to Table S5 and are labeled as follows: 1) background diversification rate, 2) BEP+PACMAD 3)Bambusoideae+Pooideae, 4) early diverging Pooideae, 5)Phaneospermateae, 6) *Perrierbambus*+*Bonia* clade 7) Poeae 2 clade, 8) *Poa*+*Alopecurus* clade, 9) *Agrostis*+*Calamagrostis* clade, 10) *Festuca*, 11) Core Panicineae, 12) Andropogoneae+Paspaleae, 13) *Sorghum*+*Andropogon* clade, 14) *Axonopus*+*Paspalum* clade, 15) *Poecilostachys*, 16) *Eragrostis* clade, 17) *Spartina* clade, 18) *Tripogon* (Table S5).

The distinctiveness of several C_4_ lineages was also highlighted by turboMEDUSA (Figure 3, Table S5). Under the macrofossil dating hypothesis, when the missing species diversity was distributed by genus, the inferred diversification rate was low in early diverging grass lineages (0.036 spp/my), increased in the common ancestor of the BEP and PACMAD clades (0.143 spp/my and further in a derived clade (core Panicoideae) containing 14–16 C_4_ origins (0.220 spp/my; Figure 3). Within the PACMAD lineage, there were an additional five accelerations in diversification rate, four of which occurred within C_4_ clades, and the fifth occurring slightly before two subsequent C_4_ origins.

**Figure 4:**
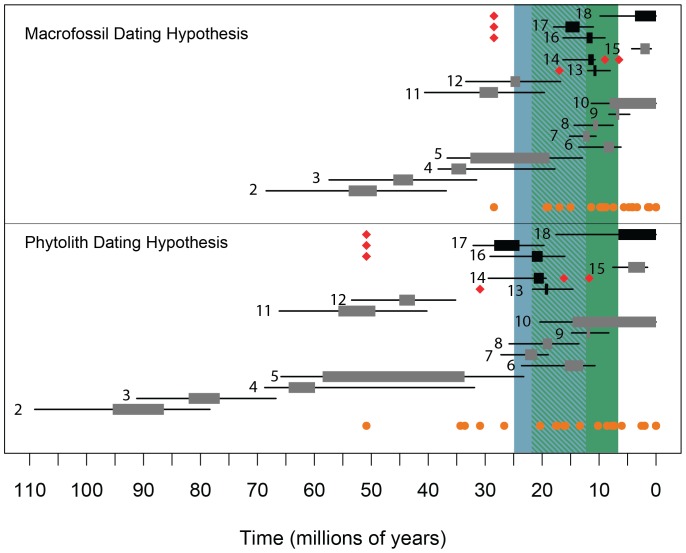
Timing of shifts in diversification rate across both dating hypotheses. Both grey and black rectangles indicate shifts in diversification rate bounded by the estimated stem and crown node ages for the branch where the shift occurred. Error bars are determined by the 95% confidence interval for each age estimate. Each shift is numbered are corresponds to the shifts in both Figure 3 and Table S5. The black rectangles indicate accelerations that occurred within C_4_ clades or immediately before C_4_ origins. Orange dots indicate the crown node ages for each of the estimated 24 origins of C_4_ photosynthesis in Poaceae. The red diamonds are the origins that are associated with subsequent rate shifts. The blue area indicates the time when grasslands are estimated to have arisen on various continents [55]–[62], [2], [63], and the green area is the time when C_3_ grasslands were replaced by C_4_ grasses [65]–[70], [2]. Overlap between the two is indicated by diagonal hatches.

In addition to these PACMAD radiations, turboMEDUSA also detected increases within the cold-adapted Pooideae grasses (Figure 3; Table S5). Although the fastest rate inferred for grasses was in a C_4_ genus, *Tripogon*, several other exceptionally high rates were found in the C_3_ Pooideae resulting in young and highly diverse taxa such as *Agrostis*, *Poa*, *Elymus*, and *Festuca*. These BEP clade radiations appeared to be concurrent with many of the warm-climate C_4_ radiations (Figure 4). The alternative dating hypothesis based on phytoliths identified precisely the same shifts, but all of the rates were slower and the timing of the shifts was earlier (Table S5, Figure 4).

The turboMEDUSA results from our 3595-tip tree (not including any missing species) were generally similar although the exact location of many of the shifts differed significantly (Table S6). In all, 12 shifts in diversification rate were identified in the full tree. There was a clear acceleration from a slow background rate (0.009 spp/my) into the BEP+PACMAD clade (0.164 spp/my), followed by a series of more recent increases. In the PACMAD, there were 5 accelerations, 1 of which occurred within the entirely C_4_ lineage Andropogoneae, and 3 of which were nested within C_4_ genera (*Muhlenbergia* and *Paspalum*). The final PACMAD acceleration was at the base of the C_3_ Danthonioideae, a lineage known, like the Pooideae, for its prominence in cool-climate grasslands [9]. In the BEP clade, accelerations within the Bambusoideae and Pooideae were followed by even more rapid diversification in *Festuca*, the Stipeae, and within *Arundinaria*.

## Discussion

### C_4_ photosynthesis promotes elevated diversification rates

In this study, we have approached the effect of C_4_ photosynthesis on diversification rates by employing a variety of statistical tests that evaluate diversification patterns in very different manners. The BiSSE analysis provides strong statistical evidence that photosynthetic type influences diversification and that across the entire Poaceae tree, C_4_ lineages have radiated faster than C_3_ lineages. This evidence is compelling, but it does not take into account variation in clade-specific diversification that may be unrelated to C_4_ photosynthesis and entirely dependent on other factors (*e.g.* generation time, dispersal strategies). However, our sister group comparisons confirmed that C_4_ lineages are statistically more speciose than their respective C_3_ sister clades. This indicates that within any given lineage background, the evolution of C_4_ photosynthesis tends to increase the number of descendant species. The “turboMEDUSA” approach estimates the position on the phylogeny where shifts in diversification rates occurred, independent of any trait information [37]. These analyses located multiple accelerations in diversification rate across Poaceae, with many in C_4_ PACMAD clades. Taken at face value, the location of most of these identified shifts suggests that there is a significant delay between the origin of the C_4_ pathway and subsequent C_4_ radiations. A similar pattern of delayed, Miocene shifts in C_4_ grasses was found in a previous diversification study using other methods [22]. Our results are also consistent with other “delayed rise” scenarios that seem to be a pattern identified in many groups of organisms [45]–[49].

C_4_ photosynthesis is a physiological trait that has no obvious links with speciation, so how can it contribute to high diversification rates? The grass lineage is exceptionally diverse for its age, and some of the diversification rates we present here are among the highest reported in the literature under either calibration scheme (Table S5) [50], [51]. The C_4_ trait might have affected diversification by increasing speciation rates, decreasing extinction rates, or both. While our data do not statistically support one scenario over another, extinction rates are more consistently lower in C_4_ lineages across a broad sampling of alternative phylogenetic trees, and lower extinction in C_4_ lineages is generally favored, although not always strongly, by BiSSE (Table S3). We suggest that the elevated competitive ability of C_4_ plants in hot, open environments [1] allowed newly formed species to survive in a range of environments, thus lowering extinction rates. Other grass-specific traits, such as their propensity for asexual reproduction, polyploidy, and long distance dispersal of seeds by wind might have acted as species-producing mechanisms in various clades throughout Poaceae [21], [52]. Under this view, net C_4_ diversification rates are higher, because fewer C_4_ species generated by these life history mechanisms went extinct than did similarly generated C_3_ species. The protracted spread of open systems might also have promoted diversification via a gradually growing patchwork of suitable habitats that allowed for repeated allopatric speciation events. In the tropics, the C_4_ trait would have increased the likelihood of successful establishment in these open areas characterized by warm temperatures, moderate drought, and high radiation loads [9], [53].

### Environmental changes and diversification of C_4_ grasses

Interestingly, interpreting the context and environmental conditions in which the MEDUSA-identified shifts occurred depends heavily upon the tree-calibrations, and whether or not the recent phylogenetic placement of phytolith fossils is indeed accurate [32]. Under the macrofossil dating hypothesis, without phytolith evidence, C_4_ origins appear almost entirely after the Oligocene atmospheric CO_2_ drop (with the possible exception of the Chloridoideae) [54], and almost all are within the time period in which C_3_ grasslands are believed to have existed on various continents [55]–[62], [2], [63]. Under this younger dating scenario, C_4_ photosynthesis may have evolved coincidently with movement into already established C_3_-dominated grasslands. Previous authors have found evidence that C_4_ origins are correlated with shifts to drier, more open habitats, which is congruent with this scenario [9], [64]. Under this timescale, all of our pinpointed C_4_ radiations occurred between about 5 and 16 Ma, during the time period in which the fossil record indicates a rapid ecological spread of C_4_ grasses [65]–[70], [2]. This dating scenario suggests concurrent diversification and rise to dominance of C_4_ species, with rapid radiations occurring well after C_4_ origins, in open, grassy biomes.

The dating hypothesis based on phytolith evidence suggests slightly different drivers of both C_4_ evolution and of diversification. In this older scenario, at least five C_4_ origins predate the fossil record of widespread open systems. In this case, C_4_ photosynthesis might still have evolved in high-radiation, open habitats, but these areas would have been rare and fragmented across the landscape. Even less intuitive is that some of these origins would have occurred when atmospheric CO_2_ levels were very high, which would result in generally lower levels of photorespiration and a weaker selection pressure for evolution of the C_4_ pathway. Regardless, in this case, both the C_4_ and Pooid radiations would have begun around 15–25 Ma, during a time period that roughly coincides with the global appearance of open C_3_-dominated grasslands on various continents, but when C_4_ species were rare [65]–[70], [2]. It would suggest that C_4_ species diversified rapidly before they became dominant features of the landscape. Only the shift in *Tripogon* could be coincident with the ecological spread of C_4_ grasses. Interestingly, this second version of events shares some similarities to phytolith-based paleo-ecological reconstructions in North America, which suggest that substantial grass taxonomic diversity predated the late Miocene C_4_ grassland expansion by 23–27 Ma [60].

Both scenarios are reasonable, and although there is not enough confidence in the phylogenetic placement of phytolith fossils [32] to prefer the older timescale, we view the abundant grass phytolith record as a remarkable resource that promises to reveal much more about the timing of past events in grass history [60], [61], [71]. Regardless of the timeframe, we want to express the genuine uncertainty inherent in any of these point estimates of diversification rate shifts. The turboMEDUSA analyses were extremely sensitive to many variables, and a slightly different taxon sampling, AICc threshold, branch-length distribution, or distribution of missing taxa would all lead to different nodes being identified—sometimes extremely different nodes (Table S6). This makes this sort of approach to diversification analyses fairly difficult to interpret. Historical patterns of diversification across such a large number of species over such a long period of time are necessarily highly complex and varied, and it seems unrealistic to assume that there are discrete, abrupt and identifiable shifts in lineage diversification through time. That said, it seems just as unrealistic to assign one diversification rate to C_4_ grasses and a second to C_3_ grasses. What feels preferable in this latter case is that this is a more diffuse analysis, averaged across all C_4_ and C_3_ branches throughout the tree, and it is a direct hypothesis that can be accepted or rejected. The specific branches and dates of the shifts identified with turboMEDUSA will surely vary as more complete phylogenies are developed, but we suspect that the BiSSE results and sister group comparisons will prove to be quite robust.

### The effect of C_4_ photosynthesis is context dependent

Although our results indicate that C_4_ photosynthesis increased diversification, its effect varies among lineages. Not every C_4_ clade experienced higher rates of diversification, and when rate accelerations occurred, they were presumably long after the initial C_4_ origin. The delay in C_4_ grass diversification might be the result of dependence on the development of a series of other adaptations to dry, open landscapes before C_4_ grasses became highly competitive. This corresponds to previous arguments made that C_4_ photosynthesis is itself only one component in suites of characters that confer ecological success or dominance [2]. Perhaps in C_4_ lineages, the right combination of traits for rapid diversification did not emerge until after considerable time had passed since the origin. Alternatively, it is well known that the C_4_ advantage is highly context dependent [1], [72], and C_4_ grasses might not have had the opportunity to diversify before open systems expanded in the Miocene.

In addition to the PACMAD C_4_ radiations, we identified a series of concurrent non-C_4_ radiations in the BEP clade. These occurred mainly within the Pooideae, particularly in lineages with well-established cold climate tolerance and temperate zone diversity. Interestingly, none of the C_3_ accelerations occurred in lineages that occupy the same climate space as C_4_ grasses. At higher latitudes, the drought and cold tolerant Pooideae could have conceivably exploited similarly expanding open biomes, but under a cooler climatic regime where C_4_ photosynthesis is not adaptive (but see Still et al. 2014 for a new perspective on temperature tolerances of Pooid grasses [73]). This indicates that while not all diverse grass lineages are C_4_, it is primarily C_4_ clades that are able to take advantage of the warm tropical, open biomes.

### Concluding thoughts

In studies of lineage diversification and character traits, the case of C_4_ photosynthesis is exceptional. There is an unusually high number (>66) of origins, providing a rare opportunity to test replicates and to identify the C_4_ effect independent of other clade-specific adaptations [74], [4]. Our sister group comparisons provide the best possible control for aspects of evolutionary history in 12 cases, and the complementary BiSSE analysis integrates over all of the origins and all of the heterogeneity in the phylogeny. The evolutionary history of grasses is inextricably linked with climatic and ecosystem changes throughout the Miocene that resulted in the global rise of grasslands [75], [2]. The evolution of C_4_ photosynthesis has long been recognized as an essential element of the ecological success of grasses in warm, open regions [1]–[3], and here we present compelling evidence that the C_4_ pathway has also behaved as a “key innovation”, promoting elevated rates of lineage diversification during the assembly of the world's grassy biomes.

## Supporting Information

Figure S1 **Histograms of BiSSE model inferences A–F.** histograms from BiSSE analyses run on trees under dating hypothesis 1 (macrofossil), G.–N. are histograms from BiSSE analyses run on trees under dating hypothesis 2 (phytolith). All are based on the results from 100 replicated phylogenies with the missing species richness distributed either proportionally (sampling frequency) or as unresolved clades. A.–B. PACMAD, unresolved clades; C.–D. Poaceae sampling frequency; E.–F. Poaceae unresolved clades; G.–H. PACMAD sampling frequency; I.–J. PACMAD unresolved clades; K.–L. Poaceae sampling frequency; M.–N. Poaceae unresolved clades.
(PDF)

Figure S2 **LTT time plot, using the 3,595 taxon tree.** Showing the accumulation of a) all Poaceae species (black), b) C_4_ species (blue), and c) C_3_ species through time (green).
(PDF)

Table S1 **The proportion of species represented by molecular data in our phylogeny.**
(DOC)

Table S2 **The number of taxa and genes used in phylgoenetic analyses for each subclade.**
(DOC)

Table S3 **Maximum credibility tree results for each set of BiSSE model comparisons.** Bold indicates the preferred model(s).
(DOC)

Table S4 **C_4_ taxa with entirely C_3_ sister clades.** Reconstructions based on character reconstructions that assume the irreversibility of C_4_ and are consistent with previous studies [10]. Bold indicates that a C_4_ clade is at least as diverse as its C_3_ sister.
(DOC)

Table S5 **Rate shifts inferred by turboMEDUSA with a pruned-to-genus tree (553 tips).** Shifts inferred with an AIC threshold of 10.5. The shift number does not correspond to the order of the shifts, but instead match Figure 3 and Figure 4. Bold indicates an acceleration in diversification in a C_4_ lineage. Asterisks mark clades that were picked up in the full tree analysis as well (Table S6).
(DOC)

Table S6 **Rate shifts inferred by turboMEDUSA with the 3595-tip tree.Shifts inferred with an AIC threshold of 17.** The shift number does not correspond to the order of the shifts. Bold indicates an acceleration in diversification in a C_4_ lineage.
(DOC)
