# Correction: C_4_ Photosynthesis Promoted Species Diversification during the Miocene Grassland Expansion

**DOI:** 10.1371/journal.pone.0105923

**Published:** 2014-08-11

**Authors:** The PLOS ONE Staff

The figure legends for Figure S1 and Figure S2 are switched. Please see the corrected figure legends below.

## Supporting Information

Figure S1 **LTT time plot, using the 3,595 taxon tree.** Showing the accumulation of a) all Poaceae species (black), b) C4 species (blue), and c) C3 species through time (green).
(PDF)

Figure S2 ** Histograms of BiSSE model inferences A–F.** histograms from BiSSE analyses run on trees under dating hypothesis 1 (macrofossil), G.–N. are histograms from BiSSE analyses run on trees under dating hypothesis 2 (phytolith). All are based on the results from 100 replicated phylogenies with the missing species richness distributed either proportionally (sampling frequency) or as unresolved clades. A.–B. PACMAD, unresolved clades; C.–D. Poaceae sampling frequency; E.–F. Poaceae unresolved clades; G.–H. PACMAD sampling frequency; I.–J. PACMAD unresolved clades; K.–L. Poaceae sampling frequency; M.–N. Poaceae unresolved clades.
(PDF)

## References

[1] Spriggs EL, Christin P-A, Edwards EJ (2014) C_4_ Photosynthesis Promoted Species Diversification during the Miocene Grassland Expansion. PLoS ONE 9(5): e97722 doi:10.1371/journal.pone.0097722 24835188 10.1371/journal.pone.0097722 PMC4023962

