# Supplementary figures and images for: C4 Photosynthesis Promoted Species Diversification during the Miocene Grassland Expansion
Source: PLoS One. 2014 May 16;9(5):e97722. doi: 10.1371/journal.pone.0097722 (PMC4023962; doi:10.1371/journal.pone.0097722)

Figure S1

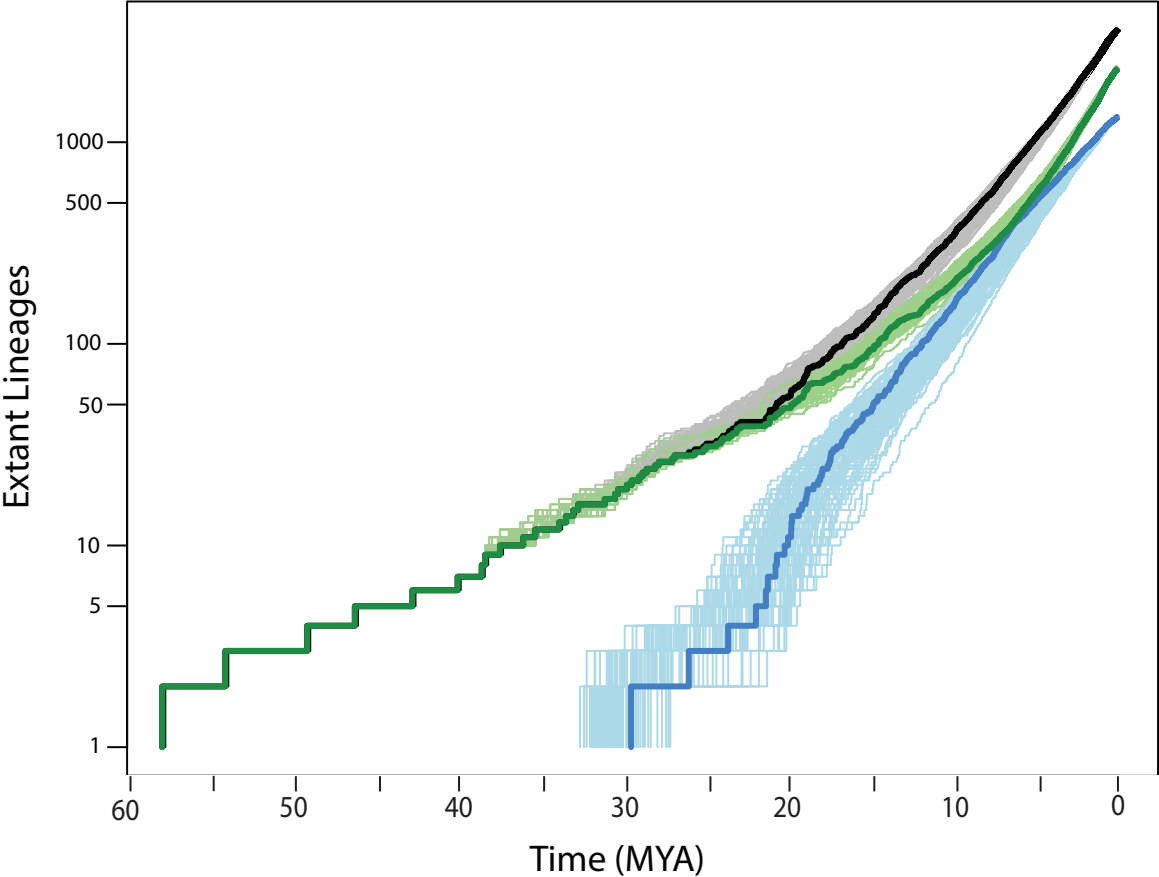

Supplement: Figure S1 — Histograms of BiSSE model inferences A–F. histograms from BiSSE analyses run on trees under dating hypothesis 1 (macrofossil), G.–N. are histograms from BiSSE analyses run on trees under dating hypothesis 2 (phytolith). All are based on the results from 100 replicated phylogenies with the missing species richness distributed either proportionally (sampling frequency) or as unresolved clades. A.–B. PACMAD, unresolved clades; C.–D. Poaceae sampling frequency; E.–F. Poaceae unresolved clades; G.–H. PACMAD sampling frequency; I.–J. PACMAD unresolved clades; K.–L. Poaceae sampling frequency; M.–N. Poaceae unresolved clades. (PDF) [file pone.0097722.s001.pdf]

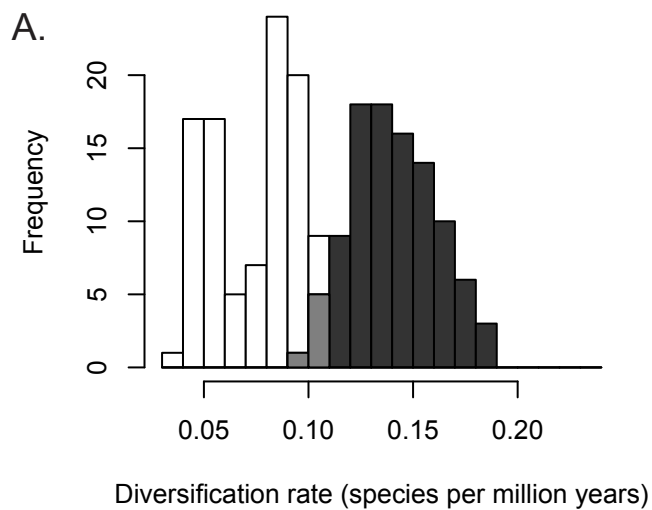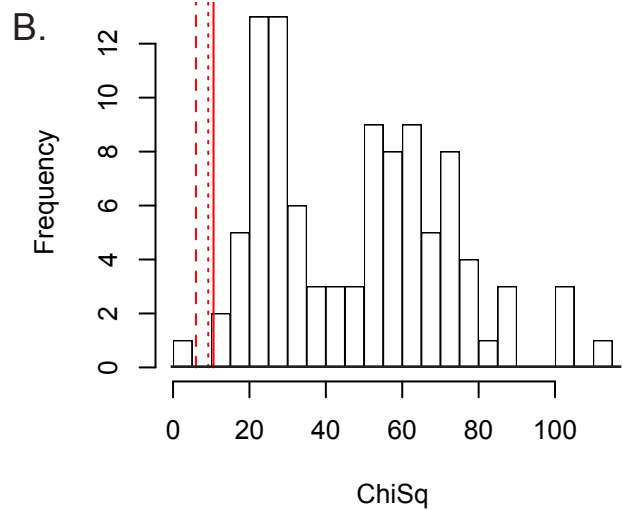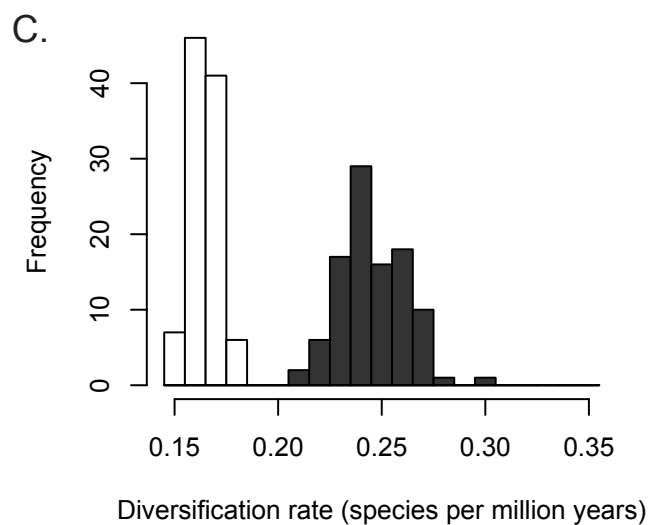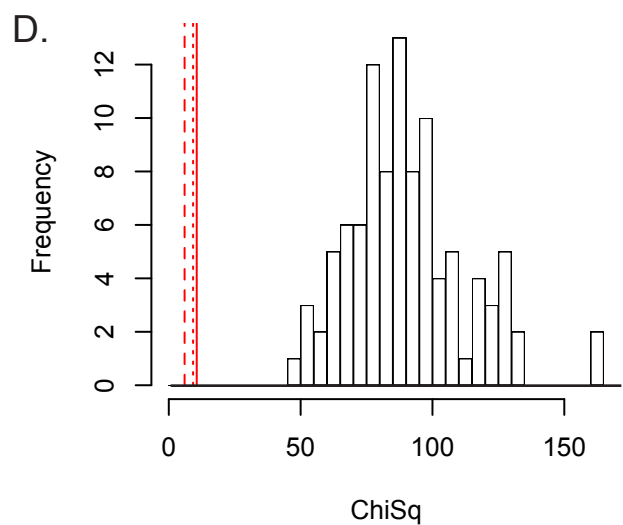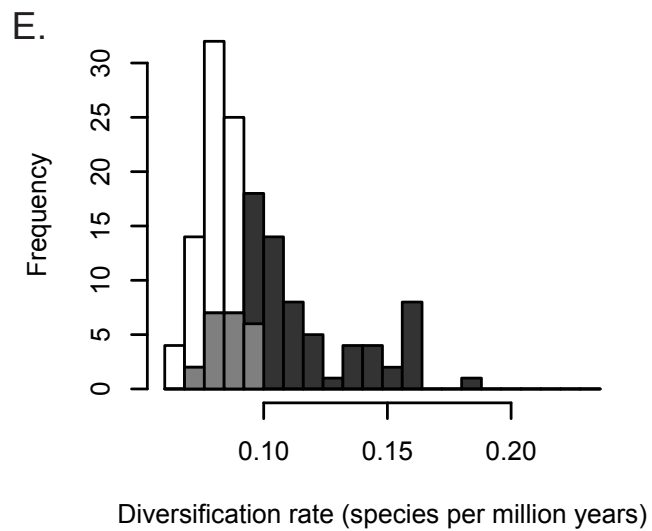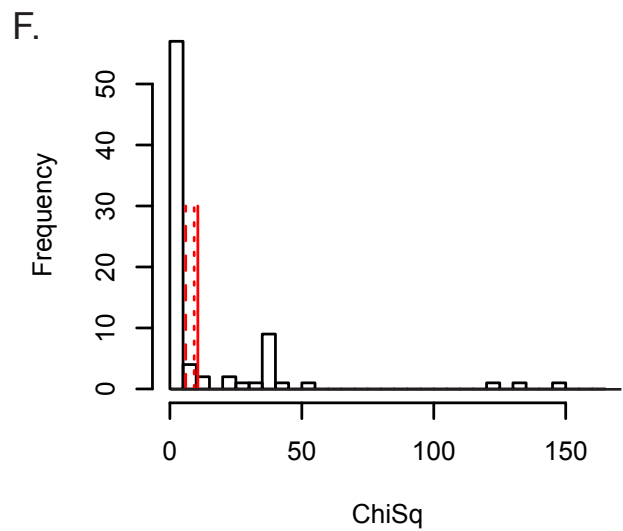

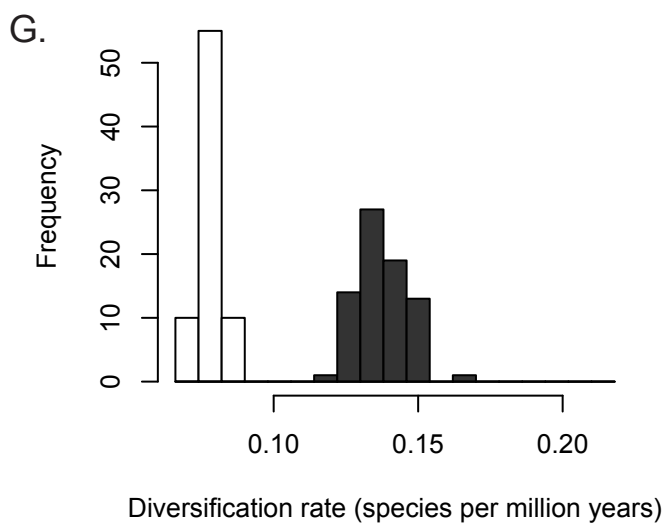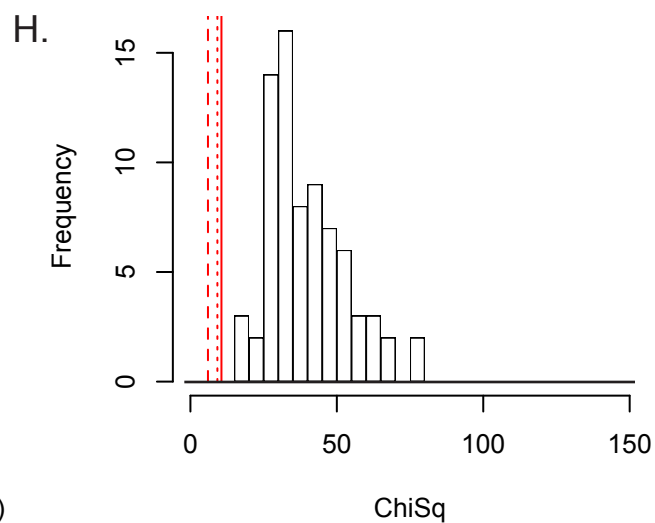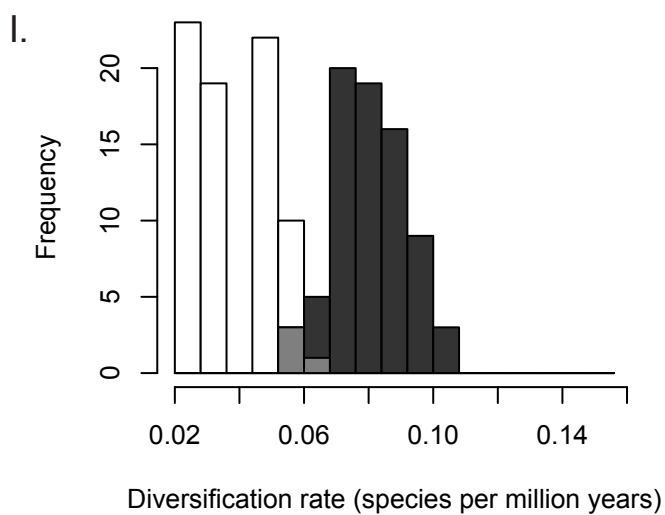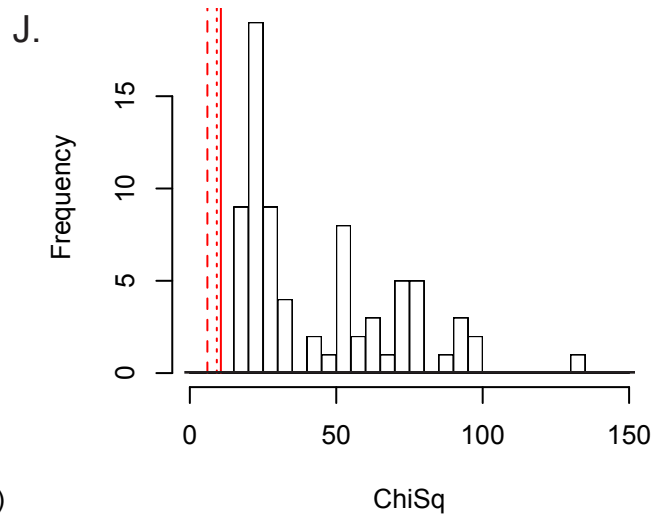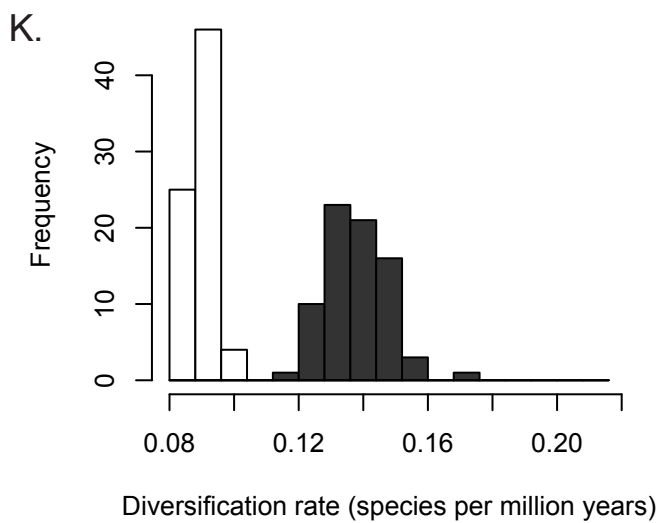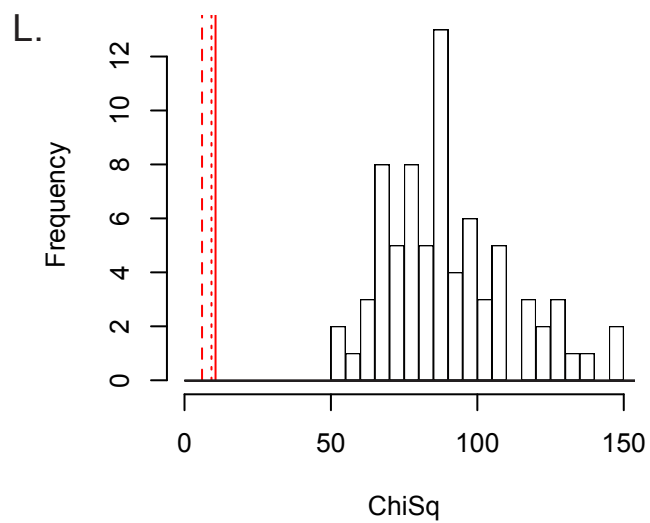

M.

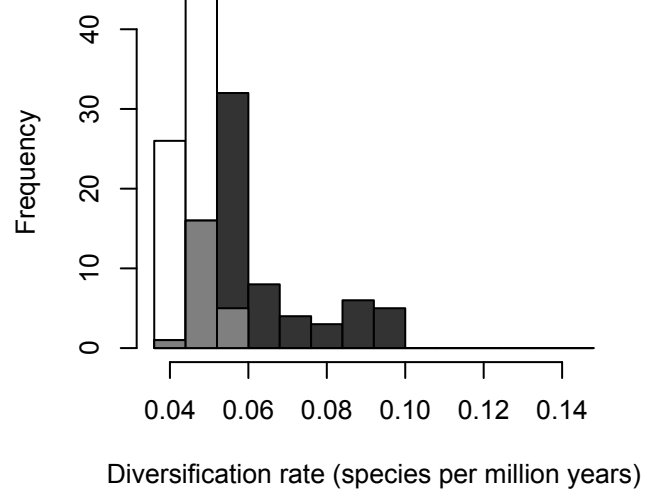

N.

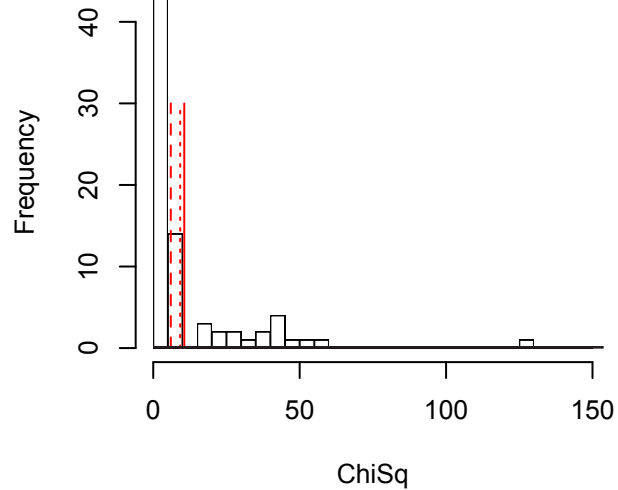

Supplement: Figure S2 — LTT time plot, using the 3,595 taxon tree. Showing the accumulation of a) all Poaceae species (black), b) C4 species (blue), and c) C3 species through time (green). (PDF) [file pone.0097722.s002.pdf]
