# Supplementary material for: C4 Photosynthesis Promoted Species Diversification during the Miocene Grassland Expansion
Source: PLoS One. 2014 May 16;9(5):e97722. doi: 10.1371/journal.pone.0097722 (PMC4023962; doi:10.1371/journal.pone.0097722)
Supplement: Table S1 — The proportion of species represented by molecular data in our phylogeny. (DOC) [file pone.0097722.s003.doc]

**Table S1.**

| Taxa | Percent in Tree |
| --- | --- |
| Poaceae | 0.2973 |
| BEP | 0.2990 |
| PACMAD | 0.2966 |
| C_3_ | 0.3173 |
| C_4_ | 0.2737 |
| PACMAD C_3_ | 0.4237 |
| PACMAD C_4_ | 0.2737 |
