# Supplementary material for: C4 Photosynthesis Promoted Species Diversification during the Miocene Grassland Expansion
Source: PLoS One. 2014 May 16;9(5):e97722. doi: 10.1371/journal.pone.0097722 (PMC4023962; doi:10.1371/journal.pone.0097722)
Supplement: Table S2 — The number of taxa and genes used in phylgoenetic analyses for each subclade. (DOC) [file pone.0097722.s004.doc]

Table S2.

| Subfamily | Species | Genes | bp | Genes |
| --- | --- | --- | --- | --- |
| Andropogoneae | 250 | 6 | 8130 | ITS,phyB,trnLtrnF,matK,ndhF,rbcL |
| Aristidoideae | 125 | 6 | 8245 | ITS,matK,ndhF,rbcL,rpL16,trnL-trnF |
| Arundinoideae- Micrairoideae | 43 | 5 | 6755 | ITS,rpoC2,matK,ndhF,rbcL |
| Bambusoideae | 419 | 11 | 13265 | matk,ndhF,rbcL,ITS,psbA-trnH,rpL16,rpL32,rps16-trnQ,trnC-rpoB,trnL-trnF,trnT-trnD |
| Centotheceae | 33 | 6 | 8515 | ndhF,rbcL,matk,trnLtrnF,phyB,ITS |
| Chloridoideae | 534 | 8 | 9152 | ITS, matK, ndhF, rbcL rl16 rps16, rps3, trnLtrnF |
| Danthonioideae | 234 | 12 | 12541 | ndhF,rbcL,atpB-rbcL,ITS,psbM,rpl16,rpoC2,trnC-trnD,trnL,ycf6-trnC,trnT-trnL,matK |
| Ehrhartoideae | 66 | 11 | 12816 | GPA1, ITS, matK, ndhC, ndhF, psbHpetB, psbZ, rbcL rps19, trnHpsbA, ycf3 |
| Paniceae | 386 | 7 | 8860 | ITS,kn1,matK,ndhF,rbcL,rpL16,trnLtrnF |
| Paspaleae | 167 | 9 | 9712 | trnL-trnF,rbcL,psbA-trnH,atpB-rbcL,trnG,rpL16,ndhF,matK,ITS |
| Pooideae | 1335 | 12 | 13144 | DMC1, ITS, matK, ndhF, pgk1, psbAtrnH, rbcL, rpb2, rpoA, rps19, trnKrps16, trnLtrnF |
| Additional taxa maintained from the backbone tree | 3 | 3 |  | matK, ndhF, rbcL |
| Total | 3595 |  |  |  |
