# Supplementary material for: C4 Photosynthesis Promoted Species Diversification during the Miocene Grassland Expansion
Source: PLoS One. 2014 May 16;9(5):e97722. doi: 10.1371/journal.pone.0097722 (PMC4023962; doi:10.1371/journal.pone.0097722)
Supplement: Table S3 — Maximum credibility tree results for each set of BiSSE model comparisons. Bold indicates the preferred model(s). (DOC) [file pone.0097722.s005.doc]

Table S3.

| **PACMAD unresolved clades** | | | |  |  |  |  |  |  |  |
| --- | --- | --- | --- | --- | --- | --- | --- | --- | --- | --- |
| **Dating Hypothesis 1** | | Speciation Rates | | Extinction Rates | | Net Diversification Rates | | Transition Rates | | Ln Likelihood |
|
| Model | Parameters | C_3_ | C_4_ | C_3_ | C_4_ | C_3_ | C_4_ | To C_3_ | To C_4_ |  |
| equal diversification | 4 | 1.6965 | 1.6965 | 1.5833 | 1.5833 | 0.1132 | 0.1132 | 0.0103 | 0.0000 | -2244.3990 |
| equal speciation | 5 | 1.6686 | 1.6686 | 1.6016 | 1.5185 | 0.0670 | 0.1501 | 0.0070 | 0.0000 | -2231.7130 |
| equal exinction | 5 | 1.6356 | 1.7260 | 1.5708 | 1.5708 | 0.0647 | 0.1552 | 0.0073 | 0.0000 | -2230.2590 |
| **full model 6 parameter** | **6** | **0.9831** | **1.9679** | **0.8880** | **1.8221** | **0.0951** | **0.1458** | **0.0113** | **0.0000** | **-2220.6650** |
|  |  |  |  |  |  |  |  |  |  |  |
| **Dating Hypothesis 2** | | C_3_ | C_4_ | C_3_ | C_4_ | C_3_ | C_4_ | To C_3_ | To C_4_ |  |
| equal diversification | 4 | 0.9383 | 0.9383 | 0.8718 | 0.8718 | 0.0665 | 0.0665 | 0.0046 | 0.0000 | -2444.5030 |
| **equal speciation** | **5** | **0.9300** | **0.9300** | **0.8962** | **0.8399** | **0.0338** | **0.0901** | **0.0038** | **0.0000** | **-2431.6390** |
| **equal exinction** | **5** | **0.8404** | **0.8964** | **0.8051** | **0.8051** | **0.0353** | **0.0913** | **0.0039** | **0.0000** | **-2428.7500** |
| **full model 6 parameter** | **6** | **0.9442** | **0.9560** | **0.9121** | **0.8689** | **0.0321** | **0.0871** | **0.0036** | **0.0000** | **-2432.0070** |
|  |  |  |  |  |  |  |  |  |  |  |
| **Poaceae Sampling Frequency** | | | |  |  |  |  |  |  |  |
| **Dating Hypothesis 1** | | Speciation Rates | | Extinction Rates | | Net Diversification Rates | | Transition Rates | | Ln Likelhood |
|
| Model | Parameters | C_3_ | C_4_ | C_3_ | C_4_ | C_3_ | C_4_ | To C_3_ | To C_4_ |  |
| equal diversification | 4 | 0.9532 | 0.9532 | 0.7618 | 0.7618 | 0.1914 | 0.1914 | 0.0027 | 0.0005 | -8911.4760 |
| equal speciation | 5 | 0.9547 | 0.9547 | 0.7556 | 0.7724 | 0.1991 | 0.1823 | 0.0028 | 0.0005 | -8910.7350 |
| equal exinction | 5 | 0.9666 | 0.9243 | 0.7546 | 0.7546 | 0.2120 | 0.1696 | 0.0029 | 0.0005 | -8905.4240 |
| **full model 6 parameter** | **6** | **1.2535** | **0.5605** | **1.0858** | **0.3199** | **0.1677** | **0.2407** | **0.0014** | **0.0008** | **-8839.8460** |
|  |  |  |  |  |  |  |  |  |  |  |
| **Dating Hypothesis 2** | | C_3_ | C_4_ | C_3_ | C_4_ | C_3_ | C_4_ | To C_3_ | To C_4_ |  |
| equal diversification | 4 | 0.5327 | 0.5327 | 0.4268 | 0.4268 | 0.1059 | 0.1059 | 0.0015 | 0.0003 | -11017.0900 |
| equal speciation | 5 | 0.5325 | 0.5325 | 0.4233 | 0.4304 | 0.1092 | 0.1021 | 0.0015 | 0.0003 | -11016.6600 |
| equal exinction | 5 | 0.5358 | 0.5140 | 0.4188 | 0.4188 | 0.1170 | 0.0952 | 0.0016 | 0.0003 | -11011.9900 |
| **full model 6 parameter** | **6** | **0.7046** | **0.3111** | **0.6131** | **0.1759** | **0.0916** | **0.1352** | **0.0008** | **0.0004** | **-10945.2500** |
|  |  |  |  |  |  |  |  |  |  |  |
| **Poaceae unresolved clades** | | | |  |  |  |  |  |  |  |
| **Dating Hypothesis 1** | | Speciation Rates | | Extinction Rates | | Net Diversification Rates | | Transition Rates | | Ln Likelihood |
|
| Model | Parameters | C_3_ | C_4_ | C_3_ | C_4_ | C_3_ | C_4_ | To C_3_ | To C_4_ |  |
| equal diversification | 4 | 3.1842 | 3.1842 | 3.0867 | 3.0867 | 0.0975 | 0.0975 | 0.0026 | 0.0002 | -3818.4140 |
| equal speciation | 5 | 3.2215 | 3.2215 | 3.1292 | 3.1184 | 0.0923 | 0.1031 | 0.0026 | 0.0002 | -3818.2990 |
| equal exinction | 5 | 3.1958 | 3.2004 | 3.1005 | 3.1005 | 0.0952 | 0.0999 | 0.0026 | 0.0002 | -3818.3940 |
| **full model 6 parameter** | **6** | **3.9240** | **1.9213** | **3.8469** | **1.7730** | **0.0771** | **0.1483** | **0.0014** | **0.0003** | **-3793.3150** |
|  |  |  |  |  |  |  |  |  |  |  |
| **Dating Hypothesis 2** | | C_3_ | C_4_ | C_3_ | C_4_ | C_3_ | C_4_ | To C_3_ | To C_4_ |  |
| equal diversification | 4 | 1.7738 | 1.7738 | 1.7184 | 1.7184 | 0.0554 | 0.0554 | 0.0015 | 0.0001 | -4146.8620 |
| equal speciation | 5 | 1.8191 | 1.8191 | 1.7668 | 1.7617 | 0.0523 | 0.0573 | 1.7617 | 0.0001 | -4146.7680 |
| equal exinction | 5 | 1.7946 | 1.7975 | 1.7408 | 1.7408 | 0.0538 | 0.0568 | 0.0015 | 0.0001 | -4146.8330 |
| **full model 6 parameter** | **6** | **2.2520** | **1.0668** | **2.2091** | **0.9822** | **0.0429** | **0.0846** | **0.0008** | **0.0001** | **-4119.9910** |
|  |  |  |  |  |  |  |  |  |  |  |
