# Supplementary material for: C4 Photosynthesis Promoted Species Diversification during the Miocene Grassland Expansion
Source: PLoS One. 2014 May 16;9(5):e97722. doi: 10.1371/journal.pone.0097722 (PMC4023962; doi:10.1371/journal.pone.0097722)
Supplement: Table S4 — C4 taxa with entirely C3 sister clades. Reconstructions based on character reconstructions that assume the irreversibility of C4 and are consistent with previous studies [10]. Bold indicates that a C4 clade is at least as diverse as its C3 sister. (DOC) [file pone.0097722.s006.doc]

Table S4.

| C_4_ lineage | C_4_ species richness | C_3_ Species richness |
| --- | --- | --- |
| *Alloteropsis* | 1 | 1 |
| ***Centropodia*** | **4** | **1** |
| **Paspaleae clade 1** | **4** | **1** |
| ***Steinchisma*** | **6** | **4** |
| **Paspaleae clade 2** | **20** | **1** |
| **Paspaleae clade 3** | **26** | **7** |
| *Eriachne* | 50 | 107 |
| ***Stipagrostis*** | **56** | **4** |
| **Outlying Panicoideae clade** | **75** | **8** |
| ***Axonopus*** | **90** | **6** |
| ***Aristida*** | **287** | **1** |
| **Panicineae clade 1** | **734** | **5** |
| *Alloteropsis* | 1 | Both C_3_ and C_4_ |
| Paspaleae clade 4 | 1 | Both C_3_ and C_4_ |
| *Reynaudia* | 1 | Both C_3_ and C_4_ |
| Panicineae clade 2 | 3 | Both C_3_ and C_4_ |
| *Anthenantia* | 4 | Both C_3_ and C_4_ |
| Paspaleae clade 5 | 4 | Both C_3_ and C_4_ |
| *Echinochloa* | 35 | Both C_3_ and C_4_ |
| Panicineae clade 3 | 282 | Both C_3_ and C_4_ |
| *Paspalum* clade | 387 | Both C_3_ and C_4_ |
| Andropogoneae | 1132 | Both C_3_ and C_4_ |
| Chloridoideae | 1527 | Both C_3_ and C_4_ |
