# Supplementary material for: C4 Photosynthesis Promoted Species Diversification during the Miocene Grassland Expansion
Source: PLoS One. 2014 May 16;9(5):e97722. doi: 10.1371/journal.pone.0097722 (PMC4023962; doi:10.1371/journal.pone.0097722)
Supplement: Table S5 — Rate shifts inferred by turboMEDUSA with a pruned-to-genus tree (553 tips). Shifts inferred with an AIC threshold of 10.5. The shift number does not correspond to the order of the shifts, but instead match Figure 3 and Figure 4. Bold indicates an acceleration in diversification in a C4 lineage. Asterisks mark clades that were picked up in the full tree analysis as well (Table S6). (DOC) [file pone.0097722.s007.doc]

**Table S5.**

| **Dating Hypothesis 1** |  |  |  |  |  |  |
| --- | --- | --- | --- | --- | --- | --- |
| Clade Name | Shift Number | Diversification Rate | Stem Node | Confidence Interval | Crown Node | Confidence Interval |
| *Background Diversification Rate | 1 | 0.0356 |  |  |  |  |
| *BEP+PACMAD | 2 | 0.1427 | 53.9827 | 40.2443-68.4535 | 49.0229 | 36.8458-60.7845 |
| *Bambusoideae+Pooideae | 3 | 0.1177 | 46.1340 | 33.8729-57.4058 | 42.6510 | 31.5072-54.0313 |
| Early Diverging Pooideae | 4 | 0.0235 | 35.9370 | 34.3299-38.2577 | 33.3416 | 17.7551-37.4408 |
| Phaenospermateae | 5 | 0.0895 | 32.6107 | 30.2748-36.6666 | 18.7226 | 12.9473-26.4088 |
| Perrierbambus+Bonia clade | 6 | 0.2451 | 9.2085 | 8.1887-13.5524 | 7.3612 | 6.1632-12.1565 |
| Poeae 2 clade | 7 | 0.2712 | 12.8100 | 12.0581-15.1607 | 11.6289 | 10.5470-14.8271 |
| Poa+Alopecurus clade | 8 | 0.5693 | 11.0653 | 9.6335-14.3623 | 10.1615 | 7.5502-12.4209 |
| Agrostis+Calamagrostis clade | 9 | 0.3552 | 6.7469 | 4.3626-8.2540 | 6.4963 | 4.6242-8.2150 |
| *Festuca | 10 | 0.6848 | 8.1610 | 6.6930-11.3468 | NA | NA |
| Core Panicineae | 11 | 0.2196 | 31.0013 | 21.7754-40.6146 | 27.7348 | 19.6102-36.5375 |
| *Andropogoneae+Paspaleae | 12 | 0.2175 | 25.5527 | 18.2317-33.3919 | 23.8568 | 16.7707-31.4497 |
| **Sorghastrum +Andropogon clade** | **13** | **0.2957** | **10.7899** | **8.2665-11.9654** | **10.4616** | **8.0697-11.9376** |
| ***Axonopus+Paspalum clade** | **14** | **0.3600** | **11.9034** | **11.2502-16.3269** | **10.9295** | **10.7146-15.7702** |
| Poecilostachys + Oplismenus | 15 | <0.0001 | 2.7574 | 1.4729-4.2638 | 1.0947 | 0.8400-2.8406 |
| **Eragrostis clade** | **16** | **0.1607** | **12.2017** | **10.1893-16.3029** | **11.1355** | **8.9745-14.4889** |
| **Spartina clade** | **17** | **0.1165** | **15.9055** | **13.7292-17.9668** | **13.4108** | **11.0246-18.3119** |
| **Tripogon** | **18** | **0.6968** | **3.7106** | **3.7075-9.8225** | **NA** | **NA** |
|  |  |  |  |  |  |  |
| **Dating Hypothesis 2** |  |  |  |  |  |  |
| Clade Name | Shift Number | Diversification Rate | Stem Node | Confidence Interval | Crown Node | Confidence Interval |
| *Background Diversification Rate | 1 | 0.0202 |  |  |  |  |
| *BEP+PACMAD | 2 | 0.0787 | 95.4155 | 83.2718-109.0901 | 86.3993 | 78.3466-95.3498 |
| *Bambusoideae+Pooideae | 3 | 0.0652 | 82.0782 | 74.2838-91.1716 | 76.6074 | 66.7483-87.0313 |
| Early Diverging Pooideae | 4 | 0.0131 | 64.5283 | 61.6426-68.6955 | 59.8682 | 31.8810-67.2286 |
| Phaenospermateae | 5 | 0.0451 | 58.5556 | 54.3613-65.8385 | 33.6182 | 23.2482-47.4195 |
| Perrierbambus+Bonia clade | 6 | 0.1407 | 16.0368 | 14.2607-23.6017 | 12.8196 | 10.7334-21.1707 |
| Poeae 2 clade | 7 | 0.1435 | 23.0016 | 21.6516-27.2225 | 20.8809 | 18.9381-26.6234 |
| Poa+Alopecurus clade | 8 | 0.3172 | 19.8689 | 17.2979-25.7890 | 18.2459 | 13.5571-22.3029 |
| Agrostis+Calamagrostis clade | 9 | 0.1637 | 12.1146 | 7.8334-14.8208 | 11.6648 | 8.3032-14.7508 |
| *Festuca | 10 | 0.3785 | 14.6538 | 12.0179-20.3743 | NA | NA |
| Core Panicineae | 11 | 0.1211 | 55.7808 | 46.1998-66.1543 | 49.2701 | 40.2406-57.6415 |
| *Andropogoneae+Paspaleae | 12 | 0.1149 | 45.0960 | 37.0440-53.4404 | 42.3349 | 35.2008-44.6338 |
| **Sorghastrum +Andropogon clade** | **13** | **0.1511** | **19.5297** | **14.9624-21.6574** | **18.9355** | **14.6063-21.6072** |
| ***Axonopus+Paspalum clade** | **14** | **0.1998** | **21.4651** | **20.2870-29.4418** | **19.7087** | **19.3213-28.4379** |
| Poecilostachys + Oplismenus | 15 | <0.0001 | 4.8896 | 2.6118-7.5608 | 1.9412 | 1.4895-5.0372 |
| **Eragrostis clade** | **16** | **0.0899** | **21.8015** | **18.2058-29.1295** | **19.8966** | **16.0353-25.8883** |
| **Spartina clade** | **17** | **0.0555** | **28.4194** | **24.5309-32.1023** | **23.9619** | **19.6983-32.7190** |
| **Tripogon** | **18** | **0.4234** | **6.6299** | **6.6245-17.5505** | **NA** | **NA** |
