# Supplementary material for: C4 Photosynthesis Promoted Species Diversification during the Miocene Grassland Expansion
Source: PLoS One. 2014 May 16;9(5):e97722. doi: 10.1371/journal.pone.0097722 (PMC4023962; doi:10.1371/journal.pone.0097722)
Supplement: Table S6 — Rate shifts inferred by turboMEDUSA with the 3595-tip tree.Shifts inferred with an AIC threshold of 17. The shift number does not correspond to the order of the shifts. Bold indicates an acceleration in diversification in a C4 lineage. (DOC) [file pone.0097722.s008.doc]

**Table S6.**

| Full Tree Dating Hypothesis 1 | |  |  |  |  |  |
| --- | --- | --- | --- | --- | --- | --- |
| Clade Name | Shift Number | Diversification Rate | Stem Node | Confidence Interval | Crown Node | Confidence Interval |
| background diversification rate | 1 | 0.0087 |  |  |  |  |
| BEP+PACMAD | 2 | 0.1634 | 53.9827 | 40.2443-68.4535 | 49.0229 | 36.8458-60.7845 |
| Poeae1+Poeae 2+Triticeae  +Bromeae | 3 | 0.3508 | 25.0338 | 21.5107-28.3134 | 23.0343 | 18.5417-24.2932 |
| Bambusoideae | 4 | 0.2769 | 42.6510 | 31.5072-54.0313 | 23.2706 | 15.6706-34.3411 |
| Arundinaria+Sasa clade | 5 | 0.4288 | 7.1132 | 4.2259-9.6557 | 5.7481 | 3.3346-7.9922 |
| Poa genus clade | 6 | 0.8962 | 5.5905 | 4.1422-7.5234 | 4.7021 | 3.7431-6.6673 |
| Stipa+Nasella+Oryziopsis clade | 7 | 0.2870 | 18.9428 | 14.9451-21.8344 | 15.1771 | 12.2263-18.7073 |
| Festuca subclade | 8 | 0.9292 | 3.4156 | 2.3939-4.8672 | 2.7934 | 2.2719-4.8026 |
| **Core Andropogoneae** | **9** | **0.2528** | **13.0748** | **9.8540-5.6070** | **12.7821** | **10.1721-14.7506** |
| Danthonioideae subclade | 10 | 0.2485 | 22.9854 | 22.7543-28.7192 | 21.0439 | 21.0054-27.4289 |
| **Muhlenbergia subclade** | **11** | **0.9050** | **2.8382** | **1.6255-3.9763** | **2.4113** | **1.3735-3.3595** |
| **Muhlenbergia subclade** | **12** | **1.2128** | **1.7970** | **1.3616-2.9154** | **1.7427** | **1.2452-2.5037** |
| **Paspalum subclade** | **13** | **0.3961** | **6.5602** | **5.8319-9.0399** | **6.3894** | **5.6624-8.7573** |

| **Full Tree Dating Hypothesis 2** | |  |  |  |  |  |
| --- | --- | --- | --- | --- | --- | --- |
| Clade Name | Shift Number | Diversification Rate | Stem Node | Confidence Interval | Crown Node | Confidence Interval |
| background rate | 1 | 0.0049 |  |  |  |  |
| BEP+PACMAD | 2 | 0.1035 | 95.4155 | 83.2718-109.0910 | 86.3993 | 78.3455-95.3498 |
| Pooideae, Bambusoideae | 3 | 0.0566 | 82.0782 | 74.2838-91.1716 | 76.6074 | 66.7483-87.0313 |
| Arundinarieae | 4 | 0.2078 | 65.7503 | 47.1303-92.0149 | 17.7463 | 13.2504-23.4427 |
| Chusquea+Guadua+Neurolepis clade | 5 | 0.1461 | 35.1760 | 32.3427-40.5234 | 32.4874 | 26.0728-38.1139 |
| Bambsua+Dendrocalamus clade | 6 | 0.2660 | 13.7097 | 11.1559-19.6955 | 12.7233 | 9.6047-17.1943 |
| Pooeae1+Poeae2+Triticeae+  Bromeae | 7 | 0.1954 | 44.9507 | 38.6245-50.8395 | 41.3604 | 33.2754-43.6207 |
| Poa genus | 8 | 0.4991 | 10.0382 | 7.4378-13.5089 | 8.4431 | 6.7211-11.9718 |
| Stipa+Nasella+Oryziopsis clade | 9 | 0.1561 | 34.0136 | 26.8354-39.2057 | 27.2519 | 21.9536-33.5908 |
| Festuca subclade | 10 | 0.5176 | 6.1330 | 4.2984-8.7395 | 5.0158 | 4.0794-8.6236 |
| **Muhlenbergia subclade** | **11** | **0.5065** | **5.0712** | **2.9044-7.1046** | **4.3084** | **2.4541-6.0026** |
| **Muhlenbergia subclade** | **12** | **0.6804** | **3.2107** | **2.4329-5.2092** | **3.1139** | **2.2248-4.4735** |
| Centotheceae | 13 | 0.0480 | 55.7808 | 46.1998-66.1543 | 53.6713 | 42.5646-62.7738 |
| **Paspalum** | **14** | **0.2197** | **11.8298** | **10.5165-16.3014** | **11.5218** | **10.2108-15.7918** |
